# High sensitivity and high resolution element 3D analysis by a combined SIMS–SPM instrument

**DOI:** 10.3762/bjnano.6.110

**Published:** 2015-04-30

**Authors:** Yves Fleming, Tom Wirtz

**Affiliations:** 1 Advanced Instrumentation for Ion Nano-Analytics (AINA), MRT Department, Luxembourg Institute of Science and Technology (LIST), 41 rue du Brill, L-4422 Belvaux, Luxembourg

**Keywords:** alloy, atomic force microscopy (AFM), correlative microscopy, differential sputtering, in situ, multimodal imaging, nano-cluster, polymer blend, secondary ion mass spectrometry (SIMS), scanning probe microscopy (SPM), SIMS artefacts, sputter-induced effects, sputter rate

## Abstract

Using the recently developed SIMS–SPM prototype, secondary ion mass spectrometry (SIMS) data was combined with topographical data from the scanning probe microscopy (SPM) module for five test structures in order to obtain accurate chemical 3D maps: a polystyrene/polyvinylpyrrolidone (PS/PVP) polymer blend, a nickel-based super-alloy, a titanium carbonitride-based cermet, a reticle test structure and Mg(OH)_2_ nanoclusters incorporated inside a polymer matrix. The examples illustrate the potential of this combined approach to track and eliminate artefacts related to inhomogeneities of the sputter rates (caused by samples containing various materials, different phases or having a non-flat surface) and inhomogeneities of the secondary ion extraction efficiencies due to local field distortions (caused by topography with high aspect ratios). In this respect, this paper presents the measured relative sputter rates between PVP and PS as well as in between the different phases of the TiCN cermet.

## Introduction

With the progress of miniaturisation, driven by future needs in various fields in materials and life sciences, the 3D analysis of devices and material structures becomes increasingly challenging. As a consequence, the interest for performing bimodal or even multimodal nano-analysis has increased during the last decade [1]. In particular, nano-analytical techniques and instruments providing both excellent spatial resolution and high-sensitivity chemical information are of utmost importance for investigations at the nanoscale. Secondary ion mass spectrometry (SIMS) is a method of choice for high sensitivity analysis, including isotopic ratio measurements [2–3]. State-of-the-art SIMS imaging instruments can provide chemical 2D and 3D maps with a lateral resolution of around 50 nm [4–5]. However, several important artefacts result from the fact that conventional 3D image reconstructions do not consider the sample surface topography, because these protocols and the applied software assume a flat sample surface as well as a cube-like analysed volume [6]. In reality, samples exhibit a surface roughness, which is also changed during the ion bombardment, because parameters such as crystal orientation and the local angle of incidence of the ion beam influence local sputter yields [6]. In case the sample is constituted of different materials, the situation is worsened due to preferential sputtering phenomena. As a consequence, the produced 3D images are affected by uncertainties on the depth scale, which are more or less important. This then causes distortions in the reconstructed 3D maps of the sample. To achieve actual high-resolution SIMS 3D analyses without risking the artefacts mentioned above, we developed a scanning probe microscopy (SPM) module that we integrated into the Cameca NanoSIMS50 [6–7]. As the environment conditions are the same (i.e., the vacuum level does not change), this in situ combination of SIMS and SPM avoids artefacts such as topography changes due to surface diffusion and the interaction of the sample with reactive species used as primary ions in SIMS [8], which occur when an ex situ combination between these same techniques is used.

The aim of this paper is to illustrate the analytical potential of the combined SIMS–SPM approach by presenting several applications in the field of materials science.

## Experimental

The integrated SIMS–SPM instrument based on a Cameca NanoSIMS 50 is presented in detail elsewhere [6–7]. The sample was sputtered with a Cs^+^ primary ion beam at 16 keV impact energy, normal incidence and sample currents between 1.4 and 2.5 pA. The raster frame was set to 256 × 256 pixels. Depending on the analysis, the dwell time per pixel ranged from 5 to 10 ms/pixel.

The SPM module inside the SIMS instrument was used to perform atomic force microscopy (AFM) in non-contact mode also called nc-AFM [9]. When scanning the topography, an area four times larger than the SIMS raster image was scanned with 512 × 512 pixels, inclosing the sputtered area as well as the area surrounding the crater, such that a full 3D correlated image can be compiled. For performing the AFM measurements, typical Si tips with a backside reflex coating were used (Nanosensors PPP-NCLR, resonance frequency of 190 kHz, *C* = 48 N/m). One AFM acquisition took between 40 and 60 min. Using this mode under vacuum conditions retraces the sample topography accurately as the thin water film, which is present on the sample surface ex situ [10–11], has evaporated.

The processing of the SIMS and AFM data was performed with the in-house developed software SARINA [12]. This software allows for the accurate superposition of SIMS and AFM data based on mostly four reference points per mapping taking into account distortions in between the various AFM images and the correlation to the respective SIMS raster scans. For the 3D reconstructed volume, the recorded topographies are taken as reference maps for linearly extrapolating the *z*-position of each of the intermittent SIMS recorded voxels. SARINA was developed as a plugin for the ImageJ software [13]. The drift correction of the different recorded SIMS stacks were performed using the OpenMIMS software [14], which is widely used in the SIMS field. The 3D SIMS-AFM surface reconstructions are visualised using the SPIP™ software by Image Metrology [15], the ParaView software tool [16] as well as the MayaVI 2 software tool [17].

## Results and Discussion

### PS/PVP polymer blend

An annealed polystyrene (PS)/polyvinylpyrrolidone (PVP) polymer blend was prepared using a 75:25 (wt %) ratio of PS/PVP homopolymers. The homopolymers of PS with molecular mass *M*_w_ = 350,000 (*M*_w_/*M*_n_ = 2.05) and PVP with molecular mass *M*_n_ = 40,000 (*M*_w_/*M*_n_ = 1.03) were obtained from Sigma-Aldrich. Both polymers were diluted in chloroform. The polymer blend with a concentration of 5 mg/mL was spin-cast onto a cleaned silicon(111) wafer. The parameters for the spin-casting were 10,000 rpm/s spinning acceleration and 3000 rpm spinning speed for a time period of 60 s. The film thickness measured by AFM was found to be 150–200 nm. The film blends were subsequently annealed above the glass transition temperature of PS. They were heated up to 140 ± 5 °C and kept at this temperature in vacuum for a time period of 6 h. After this thermal treatment, the samples were allowed to cool down slowly to room temperature.

**Figure 1:**
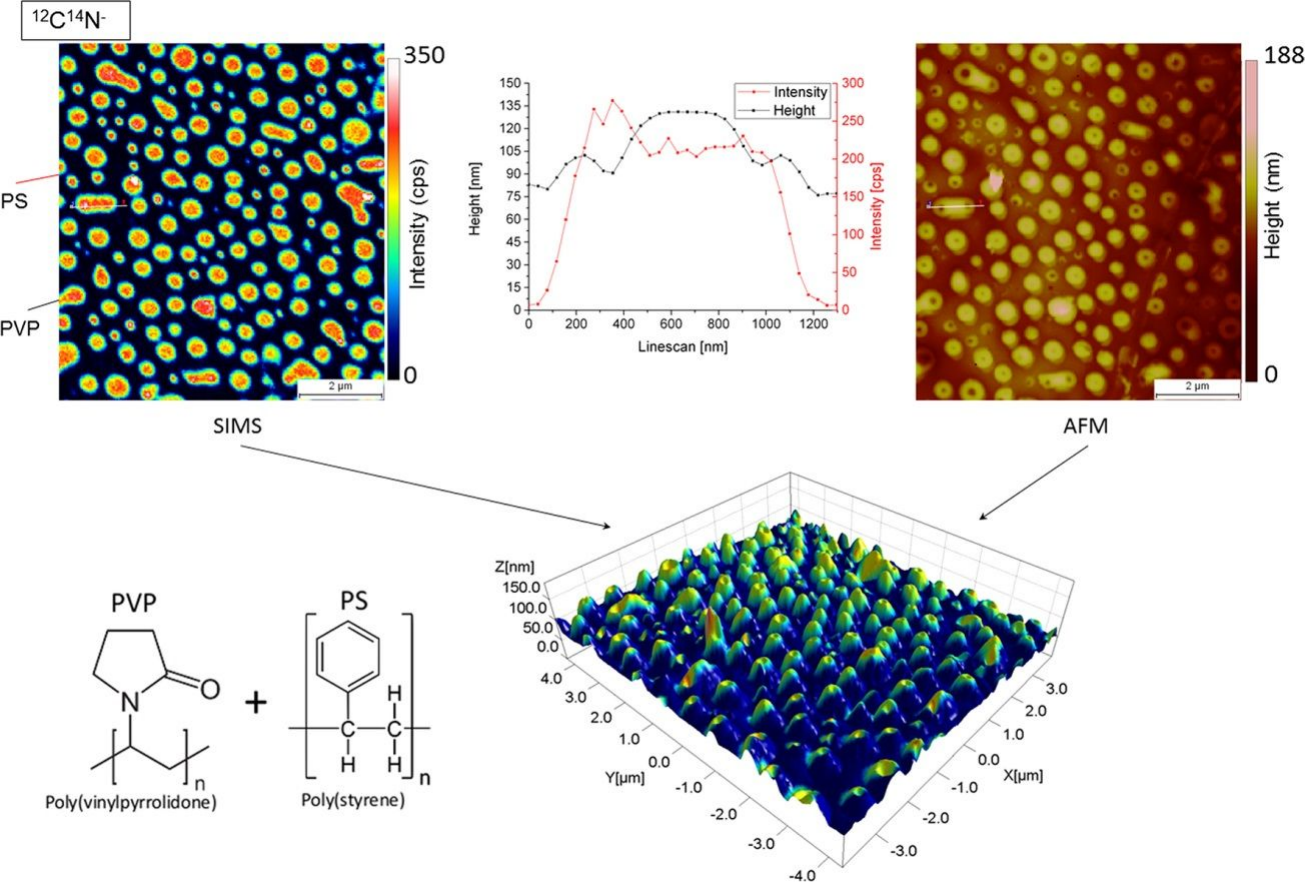
PVP/PS polymer blend after Cs^+^ bombardment of 1.02 × 10^16^ ions/cm^2^: The SIMS recorded secondary ion intensity and the AFM recorded topography of the area of interest are superposed and compiled into a 3D surface mapping.

Figure 1 shows a standard 2D SIMS image, an AFM image, the combined 3D SIMS–AFM image of the PS/PVP sample and a linescan presenting the local sample surface topography of PVP as well as the corresponding CN^−^ secondary ion signal. Because PVP contains nitrogen (in contrast to PS) its spatial distribution can be easily imaged in SIMS by tracking the CN^−^ signal. The secondary ion signal corresponding to the CN^−^ cluster is much more intense than the signal of monatomic nitrogen. The obtained 3D map shows that the two polymer phases are well separated and that the sample under investigation is far from being flat. Prior to Cs^+^ bombardment, the initial topography of the sample surface shows domes of PVP in a sea of PS. After Cs^+^ sputtering, this initial topography flattens more and more due to preferential sputtering (not shown). From topography measurements before and after SIMS analysis, it was found that the erosion rate of PVP is considerably higher than that of PS. A calculation based on the combined SIMS–AFM map leads to a sputter yield of PVP that is 3.5 times higher than the one of PS. Considering the linescan plot, it can be noticed that the secondary ion signal originating from the PVP dome is not uniform. In fact, the signal intensity is slightly increased at the position where a dip is present on the PVP dome, which is most likely due to variations of the sputtering yield with the local angle of incidence.

### Nickel-based super-alloy

**Figure 2:**
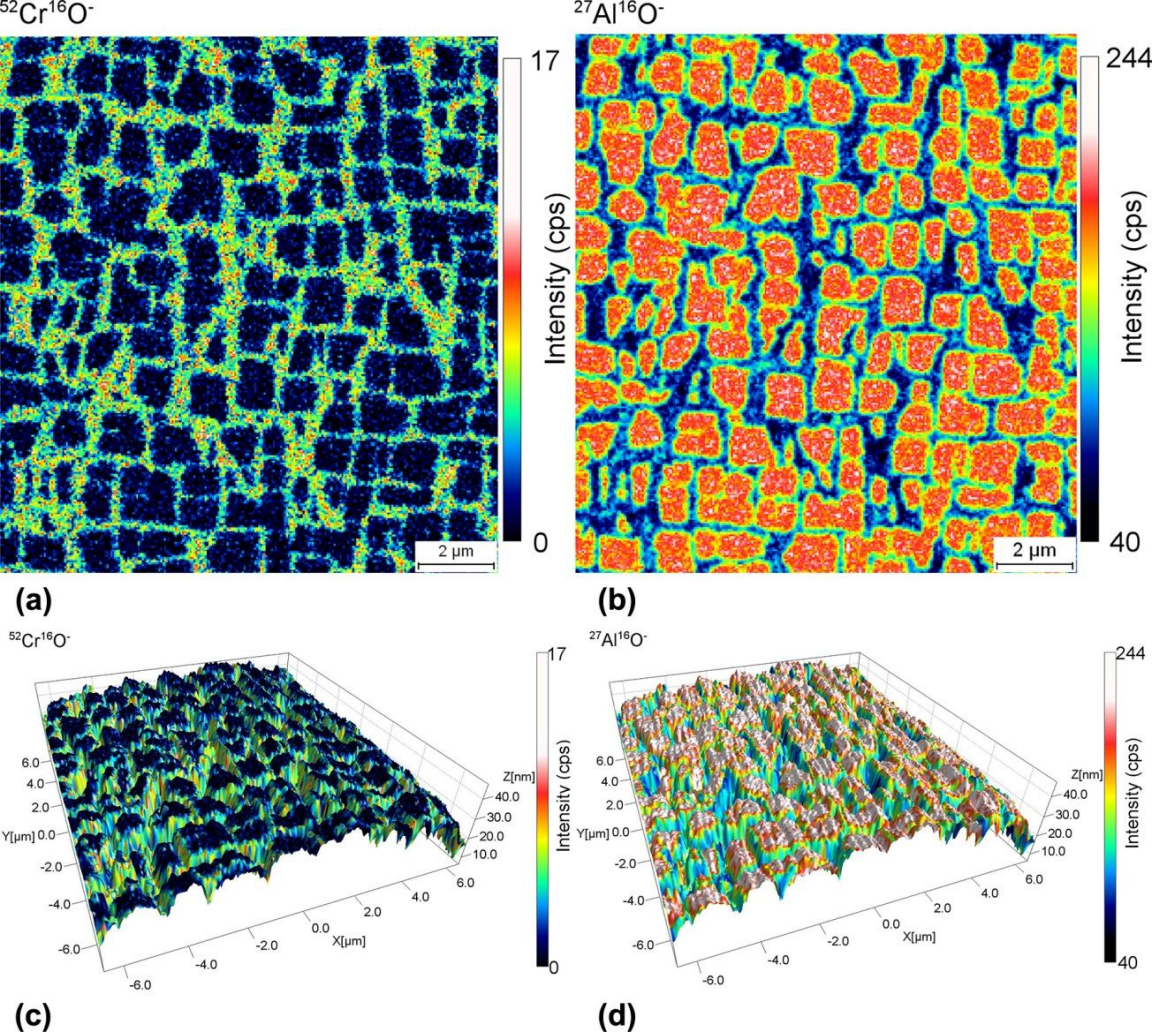
^52^Cr^16^O^−^ (a) and ^27^Al^16^O^−^ (b) secondary ion intensity recorded by the NanoSIMS instrument during the analysis of nickel-based super-alloy. The corresponding 3D SIMS-SPM reconstructions nicely show the correlations between the chemical composition and the topography (c) and (d).

Differential sputtering can also be observed when analysing nickel-based super-alloys, which are used in aerospace industry due to their high robustness and resistivity even at high temperatures [18]. The different domains inside these alloys form during the annealing step [19]. Figure 2a and Figure 2b showing the 2D SIMS images obtained on this alloy highlight that a γ′-Ni_3_Al precipitate phase is included inside the γ matrix. Figure 2c and Figure 2d show the overlay between the AFM and the SIMS mapping after sputtering a 15 × 15 µm^2^ area for 16 h. Given that the initial sample surface was flat (root mean squared roughness of 1.1 nm), it can be noticed that the aluminium-containing γ′ phase sputters much more slowly than the chromium-containing areas. The ^27^Al^16^O^−^ secondary ion signal presents a dynamics of a factor of six between inside the γ′ phase (244 cps) and outside the γ′ phase (40 cps). The secondary ion signal does not drop down to zero, as some ^27^Al^16^O^−^ ions that left the steep slopes created due to differential sputtering, are captured with an apparent pixel position outside the γ′ precipitate phase. This is a consequence of significant field inhomogeneities as a result of distortion of the local electric field arising from the surface topography. As already stated in [20], both the primary beam and the trajectories of secondary ions are perturbed by these field inhomogeneities. As a result, several artefacts, including shifts in apparent pixel position and changes in intensity, are possible. Figure 2c further shows that the ^52^Cr^16^O^−^ secondary ion signal also seems to originate from the grain boundary walls of the γ′ precipitate phase. As we know from literature that ^52^Cr is not present in the γ′ phase [19], a fraction of the ^52^Cr^16^O^−^ ions originating from the faster sputtering γ matrix are first deposited at the boundary wall of the Ni_3_Al precipitate phase before finally being re-sputtered and extracted by the secondary optics of the mass spectrometer.

### Titanium carbonitride-based cermet

**Figure 3:**
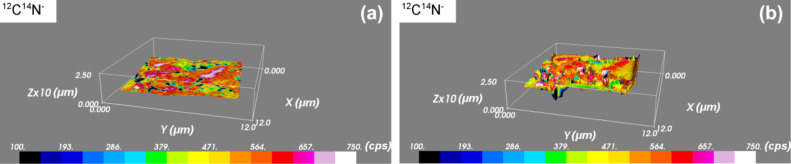
Snapshot of SIMS-SPM reconstructed surface before (a) and during (b) SIMS analysis performed on Ti(C,N). The colour scale represents the ^12^C^14^N^−^ secondary ion intensity recorded by SIMS. The carbon containing phase sputters more slowly than the phase comprising the Co binder.

The titanium carbonitride-based cermet consists of Ti(C,N) grains with a cobalt binder percolating the ceramic grains [21]. Figure 3 shows two snapshots of a Ti(C,N) sample analysed by combined AFM and SIMS. From Figure 3a, we can see that the surface of the analysed area of interest is initially flat (root mean squared roughness of 5.1 nm). After sputtering for 9 h with the Cs^+^ beam in SIMS mode, the sample holder was flipped into AFM mode again and a topography image was recorded (Figure 3b). From the combined SIMS–AFM image reconstruction, we determined that the phase containing the Co binder is sputtered at a rate of 0.28 nm·μm^2^·pA^−1^·s^−1^, compared to a sputtering rate of 0.10 nm·μm^2^·pA^−1^·s^−1^ of the Ti domains. Thus, the Co binder material is sputtered 2.8 times faster than the Ti domains. The analysis was performed with a 1.4 pA Cs^+^ primary beam.

A video animation showing the differential sputtering of this sample slice by slice can be found in Supporting Information File 1. In this animation, the surface of the grains is taken as a reference surface. From the animation, it can be noticed that the surface roughness of the grains changes in a less pronounced way than the surface roughness at the zones corresponding to the Co binder.

### EUV reticle test structures

In the field of lithography, various test structures that mimic large extreme ultra violet (EUV) reticules are commonly used. For manufacturing the structures used in this example, a TaN layer was grown through atomic layer deposition (ALD) on a Si wafer. Subsequently, using e-beam patterning the trenches were etched into the TaN layer. After processing, the test structures were deliberately contaminated for test purposes with e-beam grown carbon, as described in [22].

**Figure 4:**
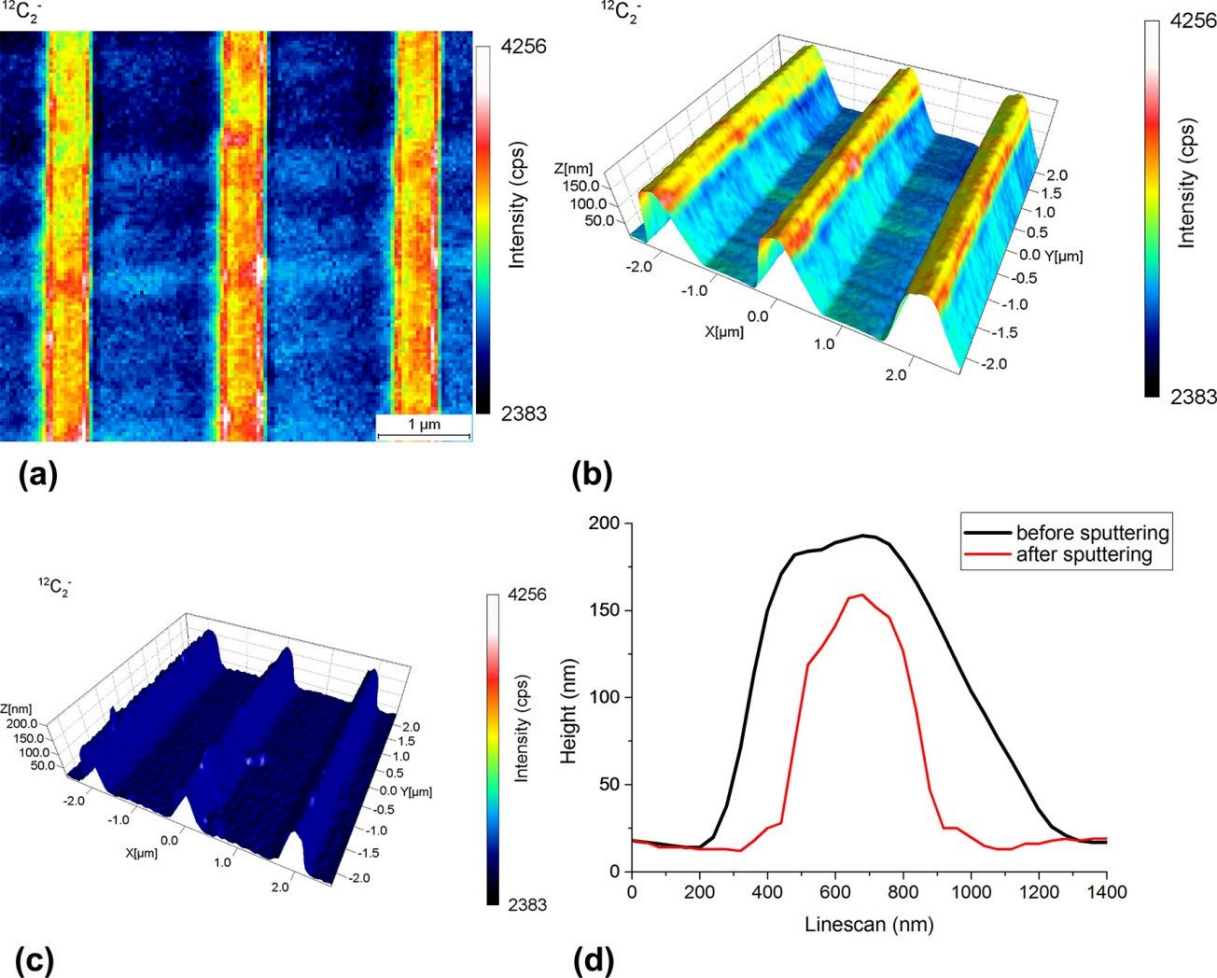
Chemical image showing the ^12^C_2_^−^ secondary ion intensity recorded from the TaN reticule with a 10 nm carbon capping layer (a). 3D SIMS–SPM reconstruction combining the chemical information as well as the topographical information after a dose of 2.4 × 10^16^ ions/cm^2^ (b) and after a dose of 1.2 × 10^17^ ions/cm^2^ (c). AFM profiles before sputtering and after sputtering with a dose of 1.2 × 10^17^ ions/cm^2^. As the trench was sputtered as well, it was preferred to plot the trench surface before and after sputtering to the same height. In this way, the change of the ridge shape is shown more clearly (d).

In Figure 4a, a SIMS image of the TaN reticule sample is shown. Here, it can be noticed that the secondary ion intensity of ^12^C_2_^−^ varies regularly although a uniform capping layer of 10 nm was deposited on top of the sample surface. The variation of the ^12^C_2_^−^ signal amounts to a factor of 1.67 between the different observed stripes. Scanning the region of interest by AFM and combining both the SIMS and the AFM data, it becomes apparent that the increased ion intensity signal is due to the fact that the analysed sample is not flat. In fact, these topographical effects are due to the curvature of the electrical field on the sample surface, resulting in a reduced extraction efficiency of the secondary ions from the trenches. With the help of the AFM topography image, recorded at the exact spot of the SIMS analysis, this SIMS artefact can be explained and thus help the analyst with the interpretation of the SIMS analysis results. Looking more carefully at the results obtained, it can be noticed that the ^12^C_2_^−^ signal falls off very sharply at the ridge edges. This sharp drop in secondary ion intensity is somewhat astonishing considering that the carbon concentration was deposited homogeneously over the sample surface. In that specific case, one would expect that the change in signal would be more gradual. Hence, the conclusion must be that more carbon was deposited on the ridge than inside the trench. The presence of carbon inside the trench can be confirmed as the originating ion signal is about 2400 cps whereas after SIMS analysis this signal has decreased to 15 cps. Indeed, the 3D SIMS–SPM reconstruction shown in Figure 4b obtained after a primary ion dose of 2.4 × 10^16^ ions/cm^2^ nicely correlates the changing SIMS ^12^C_2_^−^ signal with the changing angle of the surface of the structure.

Figure 4c shows the same sample after a SIMS analysis with a dose of 1.2 × 10^17^ ions/cm^2^. The carbon layer was now completely removed by sputtering. As can be seen from the overlaid line scans taken before and after the end of the SIMS analysis and presented in Figure 4d, it is clearly noticeable that the topography of the TaN structure is changing. Due to the higher impact angle of the primary ion beam on the ridge’s edge compared to the ridge’s top surface or the trenches, the erosion rate is considerably higher in this area. The ridges therefore become narrower and the aspect ratio of the structure is not preserved during the SIMS analysis.

### Mg(OH)_2_ nanoclusters incorporated inside a polymer matrix

**Figure 5:**
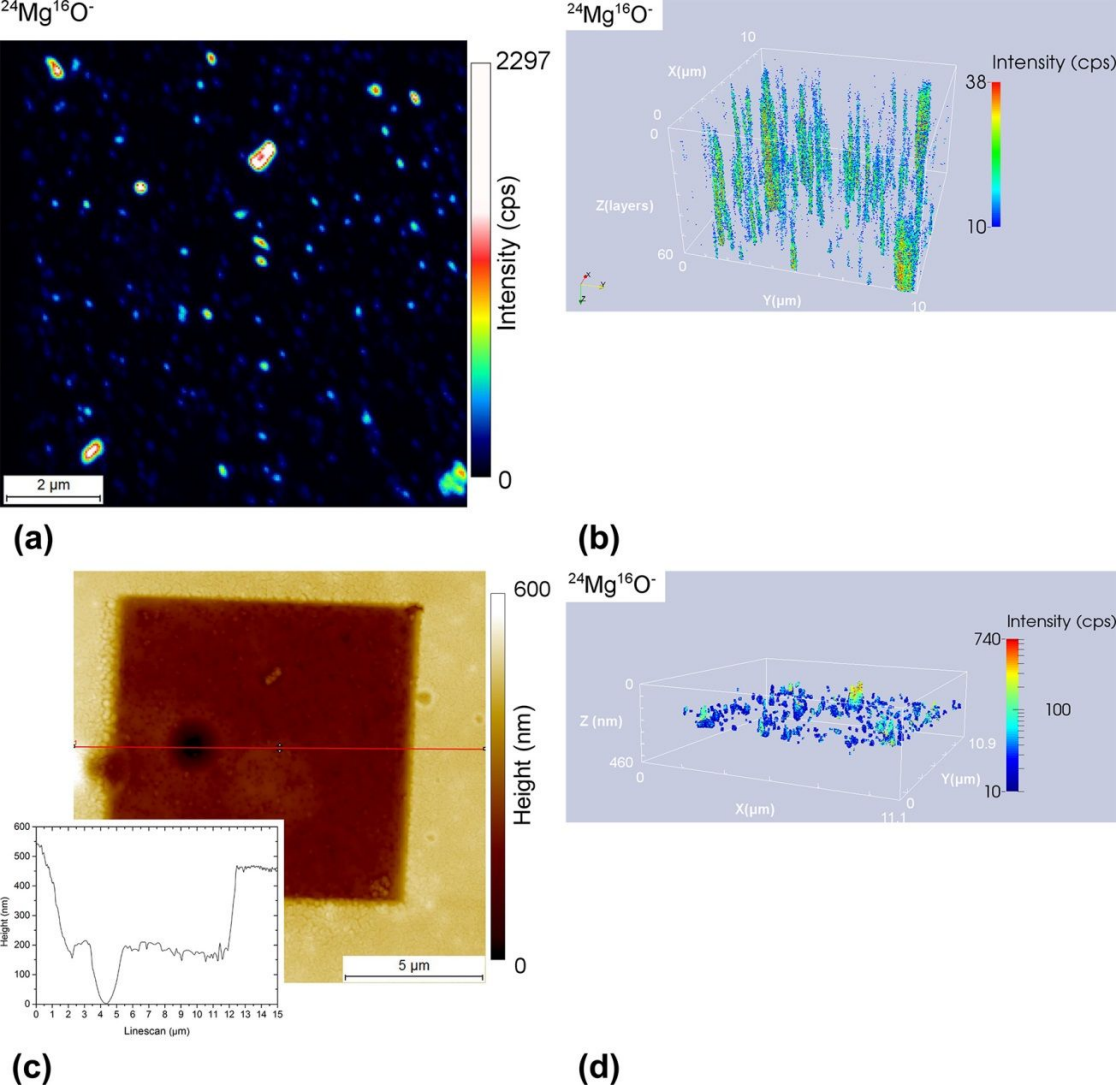
2D mapping of ^24^Mg^16^O^−^ secondary ion signal summed over analysis depth (a). 3D volume reconstruction using the traditional method assuming a flat sample surface and a homogenous sample sputtering (b) Sample topography after SIMS sputtering including inset linescan plot illustrating the depth profile through the middle of the SIMS analysed area (c) 3D volume reconstruction making use of the combined information from SIMS and AFM (d). Panels b and d are shown at the same aspect angle.

Due to the large differences in sputter rate from analysing various materials and material phases, the accurate co-localisation of nanoparticles inside a polymer matrix or inside biological tissue is very difficult. Combining SIMS with in situ AFM performed at different stages of the SIMS analysis helps to correct the depth scale and consequently gives a more accurate visualisation of the SIMS analysed volume as compared to the traditional SIMS reconstruction of the sputtered volume. Figure 5 shows the NanoSIMS 2D mapping of the ^24^Mg^16^O^−^ secondary ion signal summed over 60 layers (a), the traditional (b) and the combined SIMS–SPM (d) 3D reconstruction of the SIMS sputtered volume as well as the sample topography image after sputtering (c) of a polymer matrix incorporating 1 wt % of homogeneously distributed Mg nanoparticles in form of Mg(OH)_2_ [23]. Similar samples produced by the same group are studied in [24]. In order to visualize the nanoparticles, the secondary ion intensity of ^24^Mg^16^O^−^ was recorded on the NanoSIMS. Overall, the SIMS analysis of a 11.1 × 10.9 × 0.46 µm^3^ area took 5.5 h. The analysis was performed with a 2.5 pA Cs^+^ primary beam.

From Figure 5a, it can be noticed that the Mg nanoparticles form clusters that range from 100 to 500 nm in diameter. Comparing Figure 5b and Figure 5d, the nanoparticles portrayed in Figure 5b seem to be strongly elongated in the *z*-direction whereas in Figure 5d, the nanoparticles look more compact, but mostly distributed in a much smaller depth range (the central third of the volume). All nanoparticles are located in the upper 327 nm deep volume section. In Figure 5b, the apparent distribution of the nanoparticles over most of the sample analysis depth (60 frames) is due to the lack of topography information in the traditional NanoSIMS 3D reconstruction. From Figure 5d it can be deduced, that due to the variation in sputtering rate between the embedded nanoparticles and the polymer matrix, the initially flat topography changes during Cs^+^ bombardment and hillocks locally form at the locations of the nanoparticles. Thus, the maximum crater depth was only reached at *x*- and *y*-coordinates where no nanoparticles were found. This example and the previous examples show that the combined SIMS–AFM instrument could also help the user to analyse complicated structures such as multilayers comprising layers with different domains where differential sputtering occurs by simply flipping between the SIMS and AFM modes in a successive manner. Performing an analysis on such complicated structures would be very time consuming if not performed in situ. Knowing the sputter rates of each single layer (from measurement or literature), the analysis parameters could be programmed in a way that an AFM measurement is performed at each interface such that an accurate 3D map can be produced from the recorded SIMS and AFM data.

## Conclusion

A correlative approach between secondary ion mass spectrometry and atomic force microscopy in a single instrument leads to 3D chemical maps with highest sensitivity and enhanced spatial accuracy. This combination of techniques is of particular interest to detect and eliminate artefacts due to inhomogeneous erosion rates caused by samples containing various materials, different phases or having a non-flat surface. Using the AFM information, the evolution of the topography as a result of the sputtering can be easily monitored and quantitatively accounted for when reconstructing 3D maps. As presented in this paper, the combined SIMS–AFM technique is particularly useful when the sample to be analysed is consisting of two very different materials, where the differential sputtering between the matrix and objects of interest is large. This is, for instance, the case when metallic nanoparticles in biological samples or polymer matrices are mapped. Hence, the example of the Mg nanoclusters embedded inside a polymer matrix nicely illustrates that the 3D reconstruction combining the chemical information from SIMS and the topography information from AFM is more accurate than the traditional SIMS 3D reconstruction.

Artefacts related to inhomogeneous secondary ion extraction efficiencies due to local field distortions caused by topography with high aspect ratios are also revealed by the combined SIMS–AFM approach. This is nicely illustrated from the analysis of a nickel-based super-alloy. Thus, a fraction of ^52^Cr^16^O^−^ ions originating from the faster sputtering γ matrix are first deposited on the boundary walls of the Ni_3_Al precipitate phase before finally being re-sputtered and extracted by the secondary optics of the mass spectrometer. While this type of artefact is much more difficult to be accounted for in a quantitative manner than artefacts related to differential sputtering, the SIMS–AFM data provides valuable additional information to the analyst for the interpretation of the SIMS results. Finally, the SIMS–AFM solution is a way of measuring and comparing sputter rates more quickly. By locally measuring the topography at given stages of the SIMS analysis, the local change in topography could be mapped. In this way, we were able to measure the relative sputter rates between PS and PVP with the conclusion that PVP sputters 3.5 times faster than PS. Furthermore, with respect to the Ti(C,N) cermet, we found that the carbon-containing Ti grains sputter 2.8 times slower than the Co binding material. This same protocol could be used for the combined SIMS–AFM analysis of a multi-layered sample by recording the topography at each one of the interfaces. In the case where a number of layers presented multiple phases with large differences in sputter rate, the volume of sputtered material could be reconstructed more accurately.

## Supporting Information

The archive in Supporting Information File 1 contains two videos (MPEG II) in which a sputtering experiment and a data reconstruction are shown. One video, TiCN_12C14N_16colors_mpeg2video.mpg, shows the “live sputtering” of Ti(C,N) cermet: The cermet’s domains consisting of Ti and carbon containing grains on one hand and Co binder material on the other hand are sputtered at different rates during continued Cs^+^ bombardment. During the analysis, consisting of 160 SIMS mappings, the ^12^C^14^N^−^ sputtered ion intensity is measured in cps. The field of view covers an area of 10 × 10 µm^2^. The other video, Mg_nanoparticles_video_mpeg2video.mpg, shows a 3D reconstruction. This reconstruction makes use of the information from SIMS and SPM and shows the accurate distribution of Mg(OH)_2_ nano-clusters located in the sputtered volume (field of view: 11.1 × 10.9 × 0.46 µm^3^). The recorded ^24^Mg^16^O^−^ intensity is shown in cps.

File 1 Animated videos of sputter experiment and data reconstruction.
